# Comparison of the Effects of Intravenous Dexmedetomidine and Remifentanil in Attenuating the Cardiovascular Response to Extubation in Patients Under General Anesthesia: A Randomized Clinical Trial

**DOI:** 10.1155/anrp/9949776

**Published:** 2025-09-25

**Authors:** Hamidreza Shetabi, Mehrdad Masoudifar, Hossein Mahjobipoor, Faezeh Poorsajad

**Affiliations:** ^1^Anesthesiology Department, Isfahan University of Medical Sciences, Isfahan, Iran; ^2^Isfahan Medical School, Isfahan University of Medical Sciences, Isfahan, Iran

**Keywords:** dexmedetomidine, extubation, hemodynamics, remifentanil

## Abstract

**Background:** Considering the importance of hemodynamic stability during tracheal tube removal in patients undergoing surgery with general anesthesia. This study was designed to evaluate and compare the impacts of intravenous dexmedetomidine and remifentanil on mitigating the cardiovascular responses associated with extubation in individuals receiving general anesthesia.

**Methods:** In this clinical study, a cohort of 90 patients scheduled for surgical procedures under general anesthesia was evenly divided into three groups of 30, receiving dexmedetomidine (0.7 μg/kg), remifentanil (0.3 μg/kg), or a placebo of normal saline. Hemodynamic metrics, such as heart rate (HR), systolic blood pressure (SBP), diastolic blood pressure (DBP), and mean arterial pressure (MAP), were assessed at baseline, prior to extubation, and at intervals of 1, 3, 5, and 10 min following extubation.

**Results:** At 1–10 min postextubation, the control group (C) had a higher HR than the dexmedetomidine (D) and remifentanil (R) groups (*p* < 0.001), there was no difference in HR between the D and R groups (*p*=0.57). There was a significant difference in SBP between D and C groups (*p*=0.09) and between D and C groups (*p*=0.047). No significant differences in DBP were found among the groups (*p* > 0.0.5). There was a notable difference in MAP changes between Groups D and C (*p*=0.010), but there were no differences between Groups D and R (*p*=0.14) and R and C (*p*=0.84). In addition, the incidence of tachycardia in Group C was significantly higher than that in other groups (*p* < 0.001). In addition, the incidence of tachycardia in Group C was significantly higher than that in other groups (*p* < 0.001).

**Conclusion:** Administering dexmedetomidine at a dosage of 0.7 μg/kg enhances hemodynamic stability during extubation and decreases the occurrence of hemodynamic disturbances and coughing when compared to remifentanil at 0.3 μg/kg and the control group. Therefore, it is regarded as the optimal option for alleviating cardiovascular responses during extubation.

**Trial Registration:** Iranian Clinical Trials Registry: IRCT20200825048515N74

## 1. Introduction

Endotracheal extubation is a common procedure in anesthesia, with complications occurring more frequently than during intubation and anesthesia induction. These complications can include hypertension and tachycardia, often caused by sympathetic reflexes activated during the extubation process [1, 2]. The process of extubation can result in generally transient negative effects, including coughing, variations in hemodynamics, and changes in respiratory function; while typically manageable for healthy patients, they pose risks for those with pre-existing conditions such as intracranial disorders, hypertension, or coronary artery disease [3–5].

Various pharmacological agents, such as local anesthetics, narcotics, beta-blockers, and calcium channel blockers, are used to manage hemodynamic fluctuations during endotracheal intubation and extubation with varying effectiveness [6, 7].

It is essential to minimize hemodynamic alterations during extubation [8]. Remifentanil, a potent *μ*-receptor agonist with rapid metabolism, is particularly beneficial due to its minimal adverse effects in patients with liver or kidney disorders and its quick recovery advantages compared to other opioids [9, 10]. The research highlights that intravenous administration of remifentanil reduces heart rate (HR) increases and severe coughing during recovery, while also lowering mean arterial pressure [8].

In addition, dexmedetomidine, a selective alpha-2 adrenergic agonist, offers sedation, analgesia, and sympathetic response mitigation without respiratory depression. Its administration has been found to suppress circulatory responses during extubation, contributing to hemodynamic stability. Given the risk of severe complications, the importance of minimizing cardiovascular responses during extubation for patients with pre-existing cardiovascular or cerebrovascular complications is increased [11–13].

Therefore, finding a pharmacological agent that ensures smooth extubation with minimal adverse effects is of paramount importance. This study aims to fill this gap by evaluating the effects of intravenous dexmedetomidine versus remifentanil on cardiovascular responses to surgical extubation.

The administration of a single dose of dexmedetomidine during the extubation phase was selected to specifically mitigate the hemodynamic response associated with extubation, given that complications associated with this phase are more common than those associated with endotracheal intubation [6].

## 2. Materials and Methods

### 2.1. Study Design

This research was structured as a randomized double-blind clinical trial carried out at the Feiz Ophthalmology Center, which is associated with Isfahan University of Medical Sciences, in the year 2024. The study received approval from the Ethics Committee of Isfahan University of Medical Sciences under the code IR.MUI.MED.REC.1399.748 (date: 2024-02-18). All participants provided written informed consent in accordance with the Declaration of Helsinki.

### 2.2. Participants

A total of 90 adult patients, aged between 20 and 60 years, who were scheduled for elective surgeries necessitating general anesthesia, were included in this study. The participants were randomly divided into three groups, with each group consisting of 30 individuals.

The inclusion criteria included patients who were candidates for elective surgery under general anesthesia with a time equal to or less than two hours, age range 20–60 years, ASA = 1.2, informed consent to participate in the study, normal body mass index (BMI = 18.5–24.99), and weight between 55 and 85 kg. Also, pregnant women; diabetic patients; patients with a history of chronic lung disease; patients with uncontrolled underlying cardiovascular diseases; baseline HR less than 60 beats per minute and systolic blood pressure (SBP) less than 90 mm Hg; patients with cerebrovascular diseases; prohibition of use of study drugs; known allergy to the anesthetic drugs studied; drug abuse; renal, pulmonary, and cardiac failure; compensatory tachycardia (more than 100 beats per minute); and users of drugs with cardiovascular effects were not included in the study. The need for reintubation within 15 min of leaving the recovery room, excessive blood and fluid shifts, respiratory depression, hypoxia, nausea and vomiting, shivering, hoarseness, the possibility of difficult intubation and airway manipulation, decreased arterial oxygen saturation, and lack of extubation conditions were considered as exclusion criteria.

### 2.3. Randomization and Blinding

Participants were allocated to one of three groups, dexmedetomidine, remifentanil, or a control group, receiving normal saline through the use of computer-generated random numbers. Throughout the study, both the participants and the investigators remained unaware of the treatment assignments.

### 2.4. Preoperative Assessment

All participants underwent a comprehensive preoperative assessment including medical history review, physical examination, and laboratory tests to confirm their eligibility for participation in the study.

### 2.5. Anesthesia Protocol

All patients received continuous monitoring including electrocardiogram, sphygmomanometer, pulse oximetry, and capnography throughout surgery, and hemodynamic variables including HR, SBP, diastolic blood pressure (DBP), mean arterial pressure, and peripheral blood oxygen saturation were measured and recorded as baseline values.

Anesthesia was induced with fentanyl (2 g/kg), selected based on standard protocols [14, 15], propofol (2 mg/kg), and atracurium (0.5 mg/kg), followed by utilizing a suitable endotracheal tube. Anesthesia was maintained with propofol (100–150 μg/kg/min).

### 2.6. Intervention Administration

Before the final surgical suture, the study drugs were administered in fixed volumes by an anesthesiologist who was not affiliated with the research team. The first group (D) received dexmedetomidine at a dose of 0.7 μg/kg, diluted in 70 mL of normal saline and infused over 10 min. After that, 1 min before extubation, an additional 3 mL of saline was administered to maintain blinding of the study. The second group (R) received 70 mL of saline over the same 10 min and then 1 minute before removing the endotracheal tube, and remifentanil at a dose of 0.3 μg/kg was adjusted to a volume of 3 mL by adding normal saline then injected. The third group (S) received 70 mL of saline over 10 min and an additional 3 mL of saline 1 min before extubation. These saline injections were crucial to ensure blinding in all groups.

It is worth noting that, given the elimination half-life of dexmedetomidine, which is 2 h [16], patients were observed following the removal of the endotracheal tube.

### 2.7. Primary Outcomes

Primary outcomes include changes in HR, SBP, DBP, and mean blood pressure at baseline (prior to extubation) and at intervals of 1, 3, 5, and 10 min postextubation.

### 2.8. Secondary Outcomes

Frequency of hemodynamic disorders including hypotension, hypertension, tachycardia, and bradycardia (change of more than 30% from baseline) [17] and postextubation complications, including postextubation cough laryngospasm, hypoxia, and hoarseness.

### 2.9. Sample Size Calculation

The necessary sample size determined through G∗Power software for repeated measures ANOVA (effect size = 0.25, *α* = 0.016, power = 80%) across three groups, presuming a standard deviation (SD) of SBP of 11.1 mmHg [18], a minimum significant difference of 0.8, a 95% confidence level, and 80% power, results in 30 patients per group. A Bonferroni correction (*α* = 0.05/3) was implemented to address multiple comparisons, thereby adjusting the significance thresholds (*p* < 0.016).

### 2.10. Method of Statistical Analysis

Statistical analysis was conducted utilizing SPSS Version 26 software following the completion of data collection. Continuous variables were expressed as mean ± SD, while categorical variables were represented as frequencies (%). The analysis employed chi-square tests, one-way ANOVA, Kruskal–Wallis tests, and repeated-measures' ANOVA with Bonferroni correction were employed to evaluate differences among groups and over time points. A *p* value of less than 0.05 was deemed significant for unadjusted analyses, while an adjusted alpha level of 0.0167 (0.05/3) was applied for Bonferroni-corrected comparisons. One-way ANOVA, Kruskal–Wallis tests, and ANOVA with repeated measures were used. A *p* value of less than 0.05 was deemed statistically significant across all analyses.

## 3. Results

**Figure 1:**
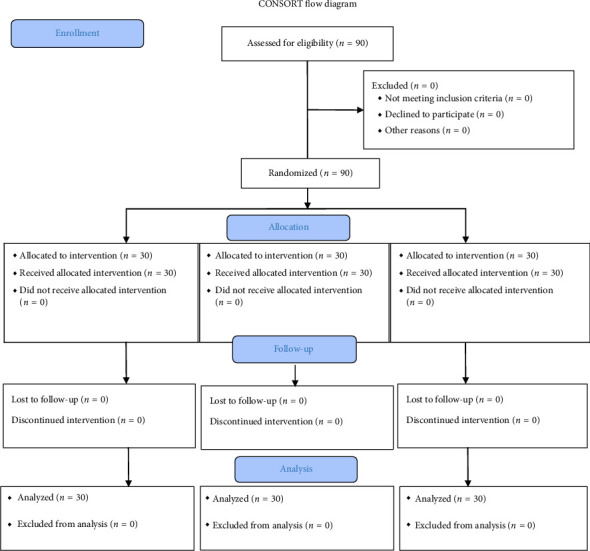
CONSORT diagram of the study.

This research involved a total of 90 patients, with each of the three groups comprising 30 participants. Group D was administered dexmedetomidine at a dosage of 0.7 μg/kg, Group R received remifentanil at 0.3 μg/kg, and Group S was given normal saline. Throughout the duration of the study, no participants were excluded due to adverse events, allowing for a comprehensive data analysis involving all 90 patients (refer to Figure 1). As indicated in Table 1, the analysis revealed no statistically significant differences among the three groups concerning demographic and clinical characteristics (*p* < 0.05).

### 3.1. Hemodynamic Parameters

No notable differences were detected in the baseline hemodynamic parameters (*p* > 0.05). Between 1 and 10 min following extubation, significant variations were observed in HR and SBP (< 0.001, Table 2). The normal saline group demonstrated elevated HR (for instance, 116.8 ± 8.8 bpm at 1 min) and SBP (145.5 ± 13.6 mmHg at 1 min) in comparison to the dexmedetomidine (84.7 ± 10.9 bpm, 129.1 ± 14.1 mmHg) and remifentanil (64.9 ± 15.6 bpm, 105.1 ± 19.1 mmHg) groups (Bonferroni-adjusted *p* < 0.001). DBP exhibited a similar trend to SBP, with significant differences noted at 1–3 min (*p* < 0.001, Bonferroni-adjusted < 0.0167) but not at 10 min (*p*=0.04, adjusted *p*=0.12). Peripheral oxygen saturation (SpO_2_) remained consistent across all time intervals (> 0.14). Repeated measure ANOVA validated significant changes within groups (*p* < 0.001) and differences between groups (*p* < 0.001).

### 3.2. Hemodynamic Disturbances

During the study period, 3, 12, and 24 patients in the three groups of dexmedetomidine, remifentanil, and normal saline developed tachycardia (10%, 40%, and 80%, respectively), and the difference between the three groups was significant (*p* < 0.001). The frequency of bradycardia in the three groups was 6, 9, and 2 cases (20%, 30%, and 6.67%, respectively), and the difference between the three groups was not significant (*p*=0.42). The frequency of hypertension was 9, 16, and 24 cases (30%, 53.3%, and 80%, respectively), and a significant difference was observed between the three groups (*p*=0.001). The frequency of hypotension in the three groups was 2, 3, and 1 cases (6.7%, 10%, and 3.7%, respectively), but the difference between the three groups was not significant (*p*=0.45).  Table 3 shows the frequency distribution of hemodynamic disorders at different times in the three groups. According to the aforementioned table, the presence of tachycardia from Minutes 1 to 10, the presence of bradycardia at Minute 1, and hypertension at Minutes 1 and 3 were significant, but at other times, there was no significant difference between the three groups. The frequency of hypotension did not differ significantly between the groups.

### 3.3. Postextubation Complications

During the study period, complications associated with extubation were observed in 28 patients, representing 31.3% of the cohort. Among these, 4 patients (13.3%) were in the dexmedetomidine group, 7 patients (23.3%) were in the remifentanil group, and 17 patients (56.7%) were in the control group. The incidence of complications varied significantly across the three groups (*p* < 0.001); notably, the frequency of cough was significantly higher in the control group than in other groups (*p*=0.031). There were no instances of severe cough reported in the dexmedetomidine and remifentanil groups; however, the control group experienced severe cough in 3 patients (*p*=0.19). Additionally, the frequency of laryngospasm (*p*=0.33), hypoxia (*p*=0.99), hoarsens (*p*=0.87), nausea (*p*=0.33), vomiting (*p*=0.33), shivering (*p*=0.32), and delirium (*p*=1) did not show significant differences among the three groups (Table 4).

## 4. Discussion

Extubation can lead to temporary adverse effects, including coughing, hemodynamic fluctuations, and respiratory changes, which are usually manageable for most patients; however, those with specific pre-existing conditions may be at greater risk.

Initial findings of the present study revealed no significant differences in demographic and clinical characteristics or baseline hemodynamic variables among the groups, suggesting that variations in outcomes were likely attributable to the type of drug administered.

Our research evaluated the effectiveness of dexmedetomidine (0.7 μg/kg) versus remifentanil (0.3 μg/kg) in reducing these responses to extubation, showing that dexmedetomidine provided better hemodynamic stability and significantly lowered the incidence of coughing compared to the control group. Additionally, there were no notable differences among the three groups in terms of laryngospasm, hypoxia, hoarseness, nausea, and vomiting rates.

Our findings align with the existing literature, highlighting the effectiveness of dexmedetomidine in managing extubation. For example, Lee et al. observed that the dexmedetomidine and remifentanil groups experienced smaller increases in systolic and DBP compared to the control group, as well as a lower HR following intubation [19].

Similarly, De Cassai et al. documented significantly lower hemodynamic responses during laryngoscopy with dexmedetomidine compared to the control group [20].

The meta-analysis conducted by Fan et al. reinforces our findings, indicating similar rates of cough between dexmedetomidine and remifentanil, while highlighting quicker recovery times associated with remifentanil [21].

A study by Shetabi et al. investigated the effects of two doses of dexmedetomidine (1 μg/kg and 0.5 μg/kg) compared to normal saline during extubation. Results showed significant reductions in SBP, mean arterial pressure, and HR in the 1 μg/kg group. The 0.5 μg/kg group also exhibited lower values than the control group at 3, 5, and 10 min's postextubation [22]. The current research indicates that administering 0.7 μg/kg of dexmedetomidine significantly mitigates hemodynamic variations following extubation by decreasing autonomic responses. Furthermore, Gupta et al.'s study involving 40 adults demonstrated that dexmedetomidine doses ranging from 0.2 to 0.7 μg/kg notably lowered HR and blood pressure postintubation in comparison to midazolam [23].

A study by Fan et al. revealed that a dose of 0.7 μg/kg of dexmedetomidine was more effective in reducing blood pressure and minimizing hemodynamic variability during laryngoscopy than a lower dose of 0.5 μg/kg. Additionally, remifentanil demonstrated greater effects than either dosage of dexmedetomidine [6]. Further research indicated that an infusion of dexmedetomidine at 0.6 μg/kg/h significantly lowered blood pressure without resulting in notable respiratory depression [24]. Moreover, administering 0.5 μg/kg of dexmedetomidine during recovery from sevoflurane remifentanil anesthesia effectively alleviated cough and hemodynamic fluctuations without exacerbating respiratory issues post-thyroid surgery [8].

The investigation revealed a significantly higher incidence of cough and respiratory depression in the control group, underscoring the importance of addressing cough severity postintubation to prevent complications. Previous research has focused on developing preventive strategies [25, 26]. Notably, Shetabi et al. found no significant difference in respiratory depression between dexmedetomidine doses and the control group; however, cough incidence was lower in the dexmedetomidine group [22].

In two separate studies by Arora and Suresh, a single administration of 0.75 μg/kg of dexmedetomidine was administered 30 min before extubation. The results showed no significant difference in sedation levels between the groups that received dexmedetomidine and those that received normal saline during the recovery period [26, 27].

Previous studies demonstrated that participants who received dexmedetomidine were easily awakened and none experienced profound sedation, so no specific intervention was required. Dexmedetomidine, an imidazole derivative, selectively binds to *α*2-receptors located in various anatomical regions, including blood vessels, sympathetic nerve terminals, the central nervous system, and the spinal cord. This *α*2-agonist promotes vasodilation, inhibits norepinephrine release, induces sedation, and contributes to analgesia. By attenuating the sympathoadrenal reflex, dexmedetomidine minimizes hemodynamic responses during extubation. Additionally, it reduces plasma epinephrine and norepinephrine levels in the perioperative period, thereby lowering overall catecholamine concentrations and inhibiting their release [28–32].

The novelty of this study lies in the direct comparison of dexmedetomidine and remifentanil at defined doses in a double-blind trial, focusing on hemodynamic stability and complications after extubation.

Limitations include the small sample size and the lack of monitoring of depth of anesthesia, which could increase the accuracy of extubation timing. Future studies should investigate larger groups and diverse surgical settings.

## 5. Conclusion

Administering dexmedetomidine at a dosage of 0.7 μg/kg offers superior hemodynamic stability during extubation when compared to remifentanil (0.3 μg/kg) and saline, effectively minimizing tachycardia, hypertension, and coughing. It is considered the preferred choice for mitigating cardiovascular reactions during extubation.

The results of the present study show that the use of a dose of 0.7 μg/kg of dexmedetomidine, compared to remifentanil and the control group, is associated with adjustment of blood pressure and HR during extubation, and patients who received dexmedetomidine had more favorable hemodynamic stability, and the incidence of hemodynamic disorders, including tachycardia, bradycardia, and increased blood pressure, was lower in this group. Therefore, it seems that the use of dexmedetomidine at a dose of 0.7 μg/kg is preferable in maintaining hemodynamic stability.

## Figures and Tables

**Table 1:** Distribution of demographic and clinical variables in three groups.

Variables	Groups			*p*
	Dexmedetomidine	Remifentanil	Normal saline	
Age (year)	44.9 ± 13.5	48.8 ± 13.1	47.9 ± 11.3	0.20
Weight (kg) (mean ± SD)	70.07 ± 10.5	70.7 ± 9.61	71.57 ± 12.89	0.83
Sex *N* (%)				
Male	18 (60)	17 (56.7)	16 (53.3)	0.87
Female	12 (40)	13 (43.3)	14 (46.7)	
ASA				
1	17 (56.7)	19 (63.3)	20 (66.7)	0.72
2	13 (43.3)	11 (36.7)	10 (33.3)	

**Table 2:** Changes in hemodynamic parameters from before extubation to 10 min after between the three groups.

Variables	Time	Groups			*p* value^∗^	Bonferroni-adjusted *p* value^∗∗^
		Dexmedetomidine (*n* = 30)	Remifentanil (*n* = 30)	Normal saline (*n* = 30)		
Heart rate (per min)	Baseline	74.9 ± 18.3	77.9 ± 14.3	75.6 ± 12.9	0.90	
	Before ext.	75.2 ± 12.2	77.9 ± 14.3	75.6 ± 11.3	0.10	
	1 min	84.7 ± 10.9	64.9 ± 15.6	116.8 ± 8.8	< 0.001	< 0.016
	3 min	82.6 ± 11.0	65 ± 13.3	109.1 ± 9.2	< 0.001	< 0.016
	5 min	80.2 ± 8.7	69.2 ± 18.7	103.8 ± 11	< 0.001	< 0.016
	10 min	78.6 ± 8.3	68.7 ± 17.3	100.2 ± 11.3	< 0.001	< 0.016

SBP (mmHg)	Baseline	126.1 ± 15.3	141.2 ± 17.3	127.3 ± 12.6	0.40	
	Before ext.	117.8 ± 15.2	125 ± 16.6	120.9 ± 12.5	0.52	
	1 min	129.1 ± 14.1	105.1 ± 19.1	145.5 ± 13.6	< 0.001	< 0.016
	3 min	125.7 ± 13.5	102.5 ± 20	142.1 ± 13	< 0.001	< 0.016
	5 min	123.7 ± 12.5	109.9 ± 19.6	132.7 ± 10.5	0.001	0.003
	10 min	123.8 ± 11.3	114.4 ± 18.7	128.8 ± 10	0.002	0.006

Diastolic blood pressure (DBP) (mmHg)	Baseline	82.1 ± 14.4	86.5 ± 12.5	78.7 ± 16.1	0.11	
	Before ext.	75.1 ± 12.3	78.4 ± 12.1	77.1 ± 10.2	0.50	
	1 min	84.5 ± 10.8	64.4 ± 17	95.2 ± 9.2	< 0.001	< 0.016
	3 min	81.6 ± 10.2	66.2 ± 13	92.7 ± 10.3	< 0.001	< 0.016
	5 min	80.3 ± 10.8	67.8 ± 14	85.6 ± 8.3	0.049	0.147
	10 min	80.3 ± 10.2	68.5 ± 14.4	82.5 ± 8.1	0.13	0.39

MAP (mmHg)	Baseline	94.3 ± 13.1	109.8 ± 13.4	95.2 ± 9.4	0.30	
	Before ext.	89.5 ± 12	98.1 ± 13.3	90.5 ± 10.5	0.93	
	1 min	98.7 ± 10.8	81.8 ± 17.4	109.8 ± 9	< 0.001	< 0.016
	3 min	95.9 ± 9.9	81.8 ± 16.4	107 ± 10	< 0.001	< 0.016
	5 min	94.4 ± 10.9	84.6 ± 16.7	100.1 ± 8.6	0.04	0.012
	10 min	94.7 ± 10.6	67.8 ± 14	94.6 ± 8.3	0.021	0.063

SpO_2_ (%)	Baseline	96 ± 1.5	96.4 ± 2.3	96.4 ± 0.98	0.14	
	Before ext.	97.03 ± 1.0	98.7 ± 1.2	97.2 ± 0.83	0.62	
	1 min	98.1 ± 0.91	99.2 ± 0.73	97.9 ± 0.76	0.65	
	3 min	98.4 ± 0.71	99 ± 1.15	98.5 ± 0.62	0.51	
	5 min	98.4 ± 0.71	99 ± 1.14	98.6 ± 0.49	0.32	
	10 min	98.3 ± 0.87	99 ± 1.01	98.6 ± 0.61	0.35	

*Note:* Variations in hemodynamic parameters among different groups and at various time points. Data are presented as mean ±  ± standard deviation.

^∗^ *p*: The unadjusted *p* value indicating differences among groups at each time point evaluated through one-way ANOVA.

^∗∗^Bonferroni-adjusted *p*: The *p* value modified for three group comparisons (*α* = 0.05/3 = 0.0167) to manage the family-wise error rate.

**Table 3:** Frequency distribution of hemodynamic disorders in three groups.

Sid effects	Time	Groups			*p*
		Dexmedetomidine	Remifentanil	Normal saline	
Tachycardia	Before ext.	1 (3.3)	2 (6.7)	1 (3.3)	1
	1 min	3 (10)	6 (20)	24 (80)	< 0.001
	3 min	0 (0)	4 (13.3)	20 (66.7)	< 0.001
	5 min	0 (0)	0 (0)	16 (53.3)	< 0.001
	10 min	0 (0)	0 (0)	13 (43.3)	< 0.001

Bradycardia	Before ext.	2 (6.7)	3 (10)	2 (6.7)	0.87
	1 min	4 (13.3)	6 (20)	0 (0)	0.002
	3 min	0 (0)	0 (0)	0 (0)	1
	5 min	1 (3.3)	3 (10)	0 (0)	0.33
	10 min	1 (3.3)	0 (0)	0 (0)	0.99

Hypertension	Before ext.	0 (0)	2 (6.7)	2 (6.7)	0.54
	1 min	9 (30)	13 (43.3)	20 (66.7)	0.001
	3 min	3 (10)	7 (23.3)	15 (50)	0.002
	5 min	3 (10)	3 (10)	4 (13.3)	0.82
	10 min	0 (0)	1 (3.3)	4 (13.3)	0.12

Hypotension	Before ext.	2 (6.7)	1 (3.3)	0 (0)	0.72
	1 min	0 (0)	1 (3.3)	0 (0)	1
	3 min	0 (0)	0 (0)	1 (3.3)	0.99
	5 min	0 (0)	1 (3.3)	0 (0)	1
	10 min	0 (0)	0 (0)	0 (0)	1

**Table 4:** Frequency types of complications in the three groups.

Kind of complication	Groups			*p*
	Dexmedetomidine	Remifentanil	Normal saline	
Cough	3 (10)	5 (16.7)	11 (36.7)	0.031
Cough severity				
No cough	27 (90)	25 (83.3)	19 (63.3)	0.19
Mild	2 (6.7)	3 (10)	5 (16.7)	
Moderate	1 (3.3)	2 (6.7)	10 (3)	
Sever	0 (0)	0 (0)	3 (10)	
Laryngospasm	0 (0)	0 (0)	2 (6.7)	0.33
Hypoxia	0 (0)	0 (0)	1 (3.3)	0.99
Hoarseness	1 (3.3)	2 (6.7)	3 (10)	0.87
Nausea	0 (0)	0 (0)	2 (6.7)	0.33
Vomiting	0 (0)	0 (0)	2 (6.7)	0.33
Shivering	0 (0)	1 (3.3)	3 (10)	0.32
Delirium	0 (0)	0 (0)	0 (0)	1

## Data Availability

The data that support the findings of this study are available from the corresponding author upon reasonable request.

## Nomenclature

D: Dexmedetomidine
R: Remifentanil
C: Control
HR: Heart rate
SBP: Systolic blood pressure
DBP: Diastolic blood pressure
MAP: Mean arterial pressure
DBP: Diastolic blood pressure
SpO_2_: Oxygen saturation
